# Supplementary material for: Integrated multi-omics analysis of the microbial profile characteristics associated with pulmonary arterial hypertension in congenital heart disease
Source: Microbiol Spectr. 2024 Oct 29;12(12):e01808-24. doi: 10.1128/spectrum.01808-24 (PMC11619245; doi:10.1128/spectrum.01808-24)
Supplement: Supplemental figures — Figures S1 to S8. [file spectrum.01808-24-s0002.pdf]

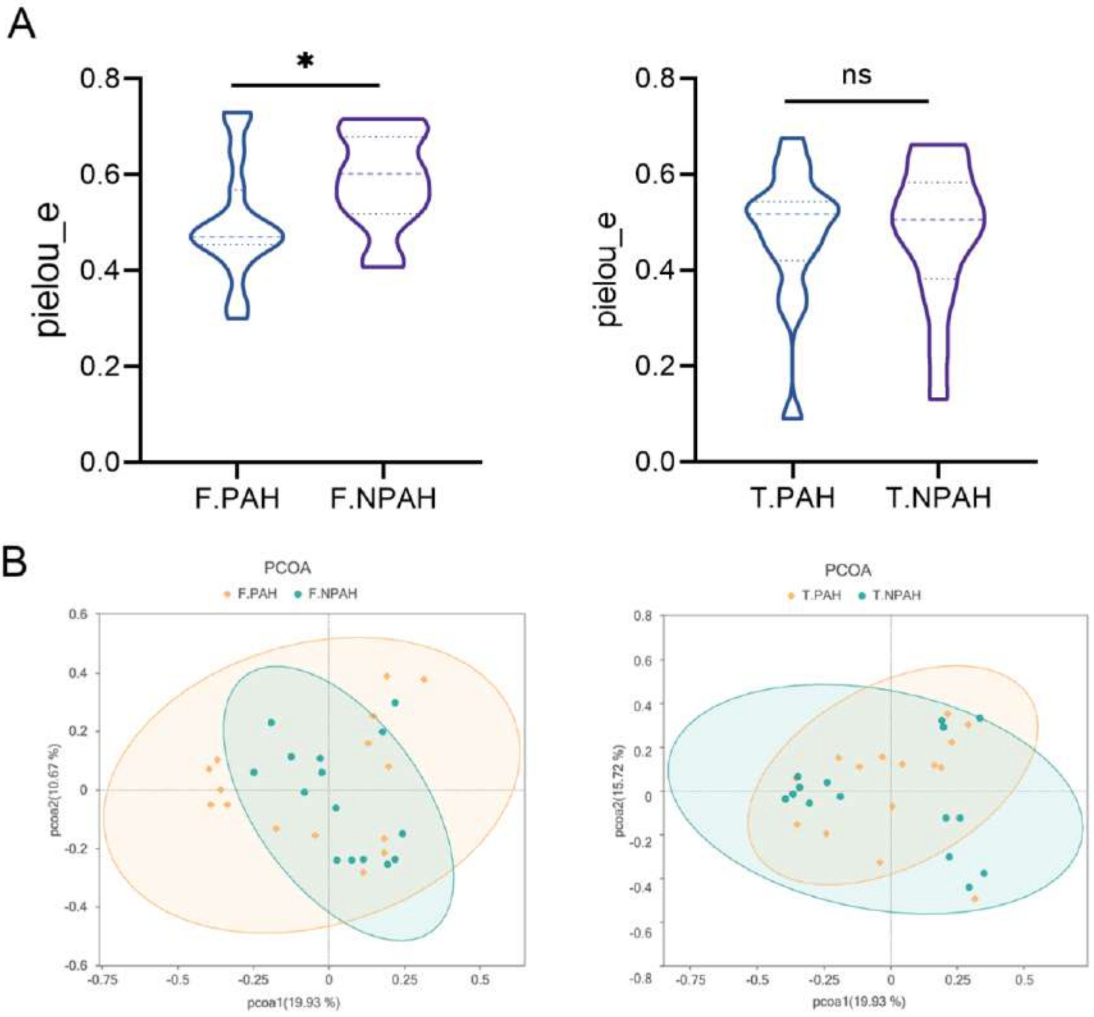

Figure S1 A,Comparison of' gut and pulmonary microbiota alpha diversity (pielou e) between thePAH-CHD and healthy controls groups.**B**, Principal Coordinate Analysis (PCoA) based on Bray-Curtis distances at the genus level shows differences between PAH-CHD and healthy controls groups (PERMANOVA). PAH: PAH-CHD groups, NPAH: Healthy controls, F-.Derived from fecal samples, T-: Derived from BLAF samples.

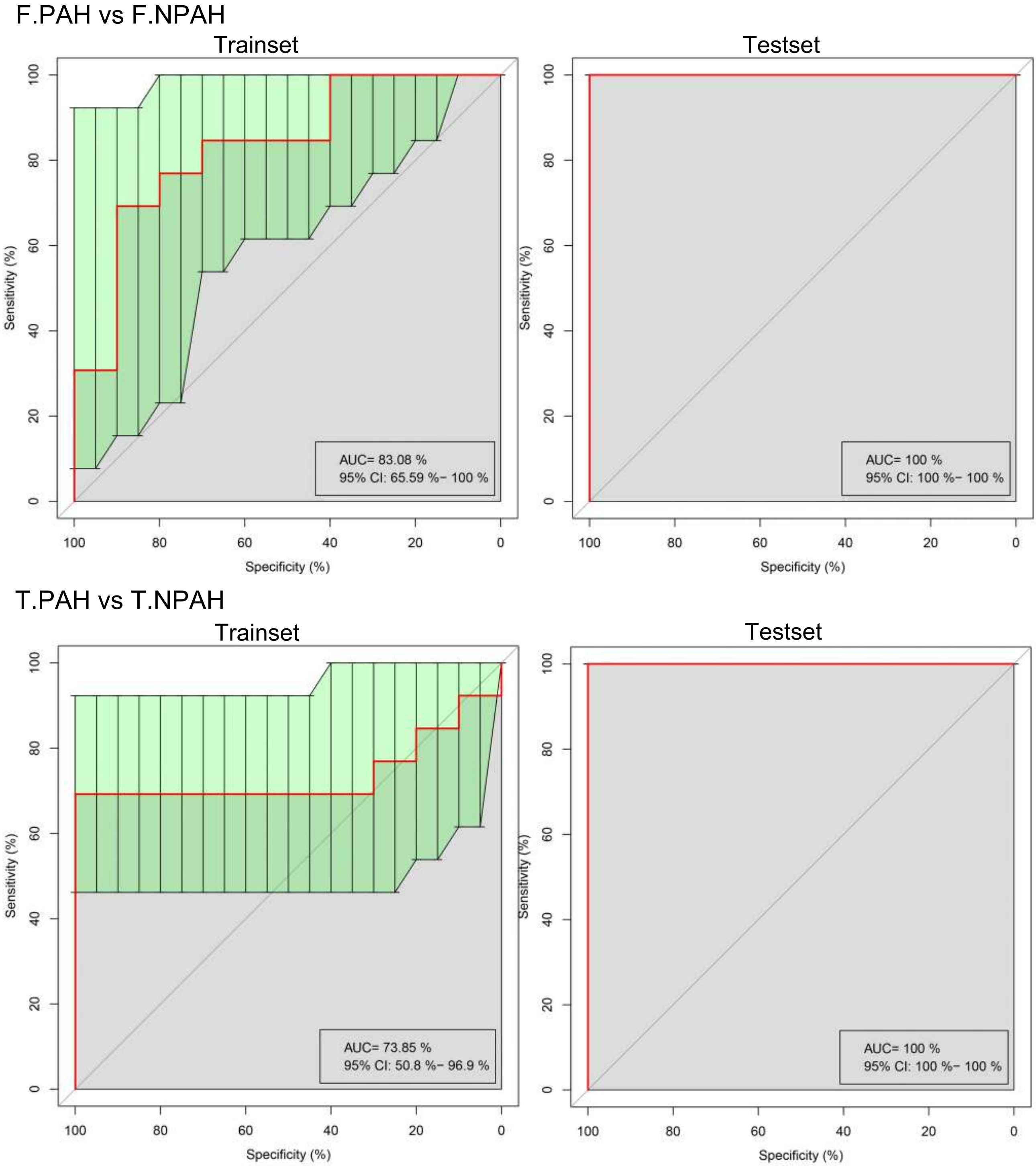

Figure S2 ROC curves plotted for cross-validation using Random Forest classifiers built with different microbiota. AUC = 0.5-0.7, indicating low accuracy; AUC = 0.7-0.8, indicating medium accuracy; AUC > 0.8, indicating high accuracy. PAH: PAH-CHD groups; NPAH: Healthy controls; F-: Derived from fecal samples; T-: Derived from BLAF samples.

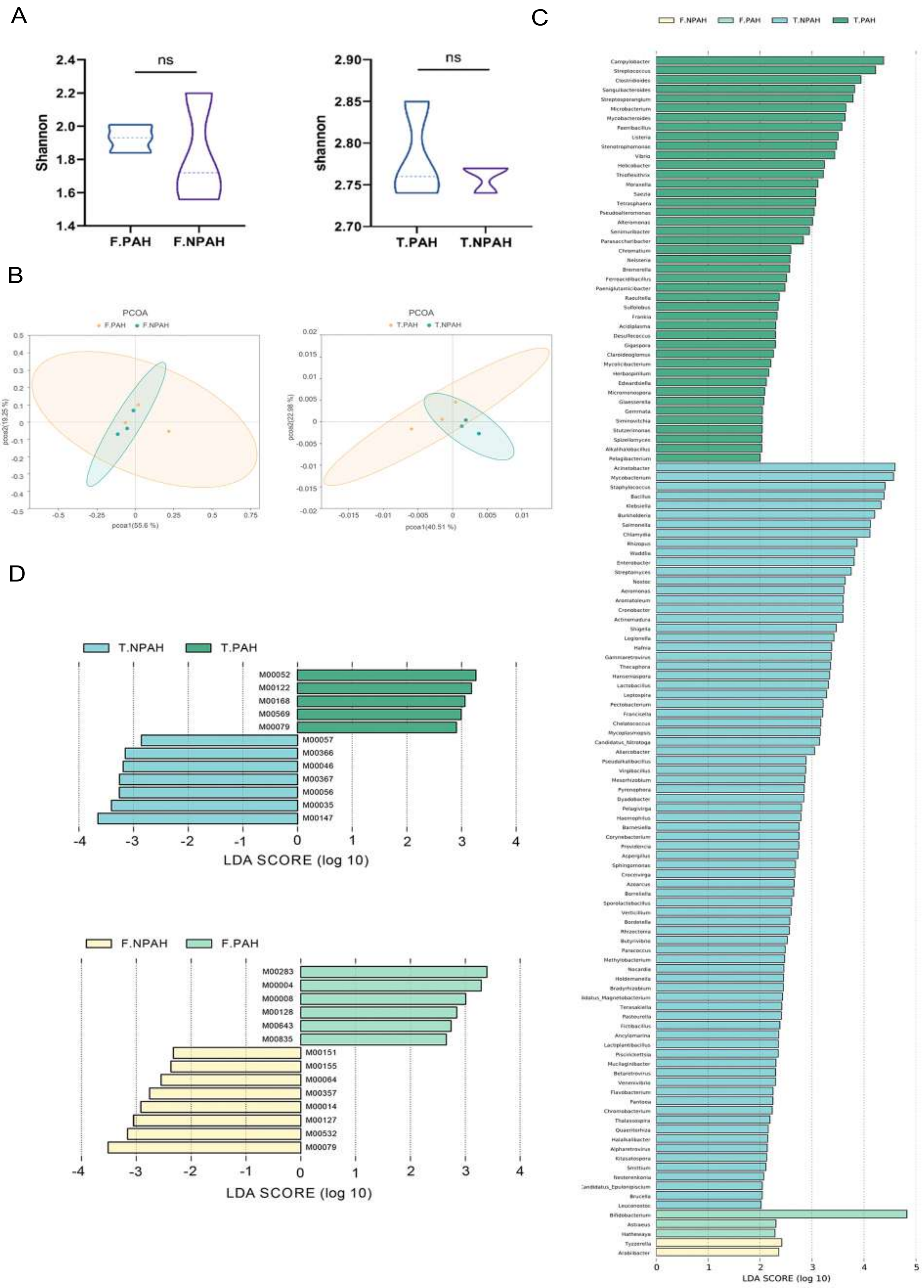
A
ns
Shannon
2.4
2.2
2.0
1.8
1.6
1.4
F.PAH
F.NPAH
ns
shannon
2.90
2.85
2.80
2.75
2.70
T.PAH
T.NPAH
B
PCOA
F.PAH
F.NPAH
pcoa1(55.6 %)
pcoa2(19.25 %)
PCOA
T.PAH
T.NPAH
pcoa1(40.51 %)
pcoa2(22.98 %)
C
F.NPAH
F.PAH
T.NPAH
T.PAH
LDA SCORE (log 10)
D
T.NPAH
T.PAH
M00052
M00122
M00168
M00569
M00079
M00057
M00366
M00046
M00367
M00056
M00035
M00147
LDA SCORE (log 10)
F.NPAH
F.PAH
M00283
M00004
M00008
M00128
M00643
M00835
M00151
M00155
M00064
M00357
M00014
M00127
M00532
M00079
LDA SCORE (log 10)

Figure S3 Metagenomic profiling of gut and pulmonary microbiota in PAH-CHD and healthy controls children. **A**, Comparison of gut and pulmonary microbiota alpha diversity between the PAH-CHD and healthy controls groups (Wilcoxon rank-sum test). **B**, PCoA based on Bray-Curtis distances of genus-level bacterial composition shows differences between the PAH-CHD and healthy controls groups (PERMANOVA). **C**, LEfSe analysis indicates differences in genus-level enriched microbiota between the PAH-CHD and healthy controls groups (LDA>2). **D**, Evolutionary chart of differentially enriched taxa between the PAH-CHD and healthy controls groups from LEfSe analysis. **E**, LEfSe analysis reveals differentially enriched KEGG modules between the PAH-CHD and Healthy controls groups (LDA>2.5). PAH: PAH-CHD groups; NPAH: Healthy controls; F-: Derived from fecal samples; T-: Derived from BLAF samples.

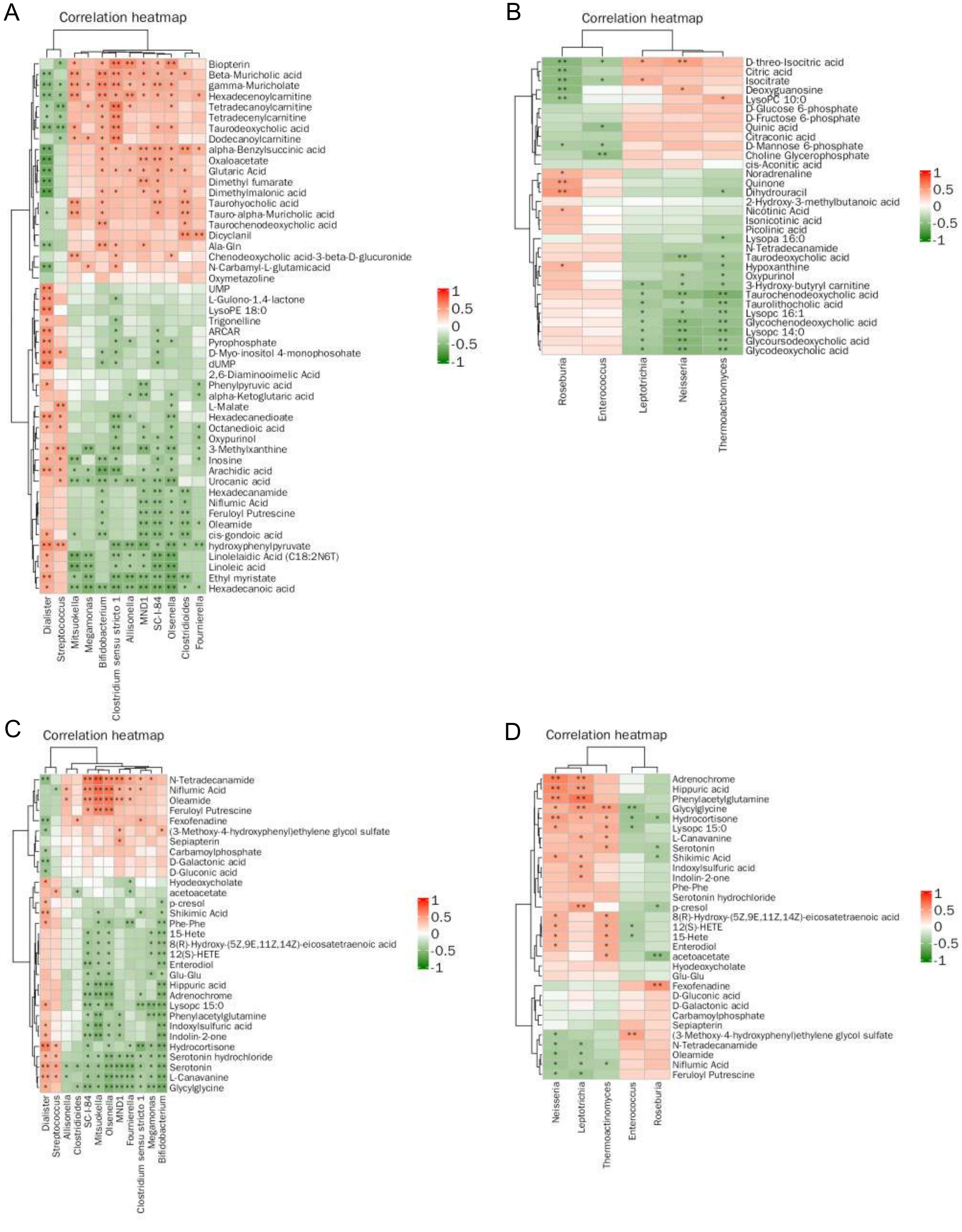
A
Correlation heatmap
Biopterin
Beta-Muricholic acid
gamma-Muricholate
Hexadecenoylcarnitine
Tetradecanoylcarnitine
Tetradecenylcarnitine
Taurodeoxycholic acid
Dodecanoylcarnitine
alpha-Benzylsuccinic acid
Oxaloacetate
Glutaric Acid
Dimethyl fumarate
Dimethylmalonic acid
Taurohyocholic acid
Tauro-alpha-Muricholic acid
Taurochenodeoxycholic acid
Dicyclanil
Ala-Gln
Chenodeoxycholic acid-3-beta-D-glucuronide
N-Carbamyl-L-glutamicacid
Oxymetazoline
UMP
L-Gulono-1,4-lactone
LysoPE 18:0
Trigonelline
ARCAR
Pyrophosphate
D-Myo-inositol 4-monophosohate
dUMP
2,6-Diaminooimelic Acid
Phenylpyruvic acid
alpha-Ketoglutaric acid
L-Malate
Hexadecanedioate
Octanedioic acid
Oxypurinol
3-Methylxanthine
Inosine
Arachidic acid
Urocanic acid
Hexadecanamide
Niflumic Acid
Feruloyl Putrescine
Oleamide
cis-gondoic acid
hydroxyphenylpyruvate
Linolelaidic Acid (C18:2N6T)
Linoleic acid
Ethyl myristate
Hexadecanoic acid
Dialister
Streptococcus
Mitsuokella
Megamonas
Bifidobacterium
Clostridium sensu stricto 1
Allisonella
MND1
SC-I-84
Olsenella
Clostridioides
Fournierella
1
0.5
0
-0.5
-1
B
Correlation heatmap
D-threo-Isocitric acid
Citric acid
Isocitrate
Deoxyguanosine
LysoPC 10:0
D-Glucose 6-phosphate
D-Fructose 6-phosphate
Quinic acid
Citraconic acid
D-Mannose 6-phosphate
Choline Glycerophosphate
cis-Aconitic acid
Noradrenaline
Quinone
Dihydrouracil
2-Hydroxy-3-methylbutanoic acid
Nicotinic Acid
Isonicotinic acid
Picolinic acid
Lysopa 16:0
N-Tetradecanamide
Taurodeoxycholic acid
Hypoxanthine
Oxypurinol
3-Hydroxy-butyryl carnitine
Taurochenodeoxycholic acid
Taurolithocholic acid
Lysopc 16:1
Glycochenodeoxycholic acid
Lysopc 14:0
Glycoursodeoxycholic acid
Glycodeoxycholic acid
Roseburia
Enterococcus
Leptotrichia
Neisseria
Thermoactinomyces
1
0.5
0
-0.5
-1
C
Correlation heatmap
N-Tetradecanamide
Niflumic Acid
Oleamide
Feruloyl Putrescine
Fexofenadine
(3-Methoxy-4-hydroxyphenyl)ethylene glycol sulfate
Sepiapterin
Carbamoylphosphate
D-Galactonic acid
D-Gluconic acid
Hyodeoxycholate
acetoacetate
p-cresol
Shikimic Acid
Phe-Phe
15-Hete
8(R)-Hydroxy-(5Z,9E,11Z,14Z)-eicosatetraenoic acid
12(S)-HETE
Enterodiol
Glu-Glu
Hippuric acid
Adrenochrome
Lysopc 15:0
Phenylacetylglutamine
Indoxylsulfuric acid
Indolin-2-one
Hydrocortisone
Serotonin hydrochloride
Serotonin
L-Canavanine
Glycylglycine
Dialister
Streptococcus
Allisonella
Clostridioides
SC-I-84
Mitsuokella
Olsenella
MND1
Fournierella
Clostridium sensu stricto 1
Megamonas
Bifidobacterium
1
0.5
0
-0.5
-1
D
Correlation heatmap
Adrenochrome
Hippuric acid
Phenylacetylglutamine
Glycylglycine
Hydrocortisone
Lysopc 15:0
L-Canavanine
Serotonin
Shikimic Acid
Indoxylsulfuric acid
Indolin-2-one
Phe-Phe
Serotonin hydrochloride
p-cresol
8(R)-Hydroxy-(5Z,9E,11Z,14Z)-eicosatetraenoic acid
12(S)-HETE
15-Hete
Enterodiol
acetoacetate
Hyodeoxycholate
Glu-Glu
Fexofenadine
D-Gluconic acid
D-Galactonic acid
Carbamoylphosphate
Sepiapterin
(3-Methoxy-4-hydroxyphenyl)ethylene glycol sulfate
N-Tetradecanamide
Oleamide
Niflumic Acid
Feruloyl Putrescine
Neisseria
Leptotrichia
Thermoactinomyces
Enterococcus
Roseburia
1
0.5
0
-0.5
-1

Figure S4 Correlations between genus-level microbiota and metabolites in PAH-CHD children. **A**, Correlation between gut microbiota and metabolites. **B**, Correlation between pulmonary microbiota and metabolites. **C**, Correlation between gut microbiota and blood metabolites. **D**, Correlation between pulmonary microbiota and blood metabolites. Spearman correlation analysis was used to determine correlations. Blue indicates positive correlation, orange indicates negative correlation. Wilcoxon rank-sum test was used, with multiple testing correction by Benjamini-Hochberg false discovery rate (FDR; $P<0.05$). *$P<0.05$, **$P<0.01$, and ***$P<0.001$.

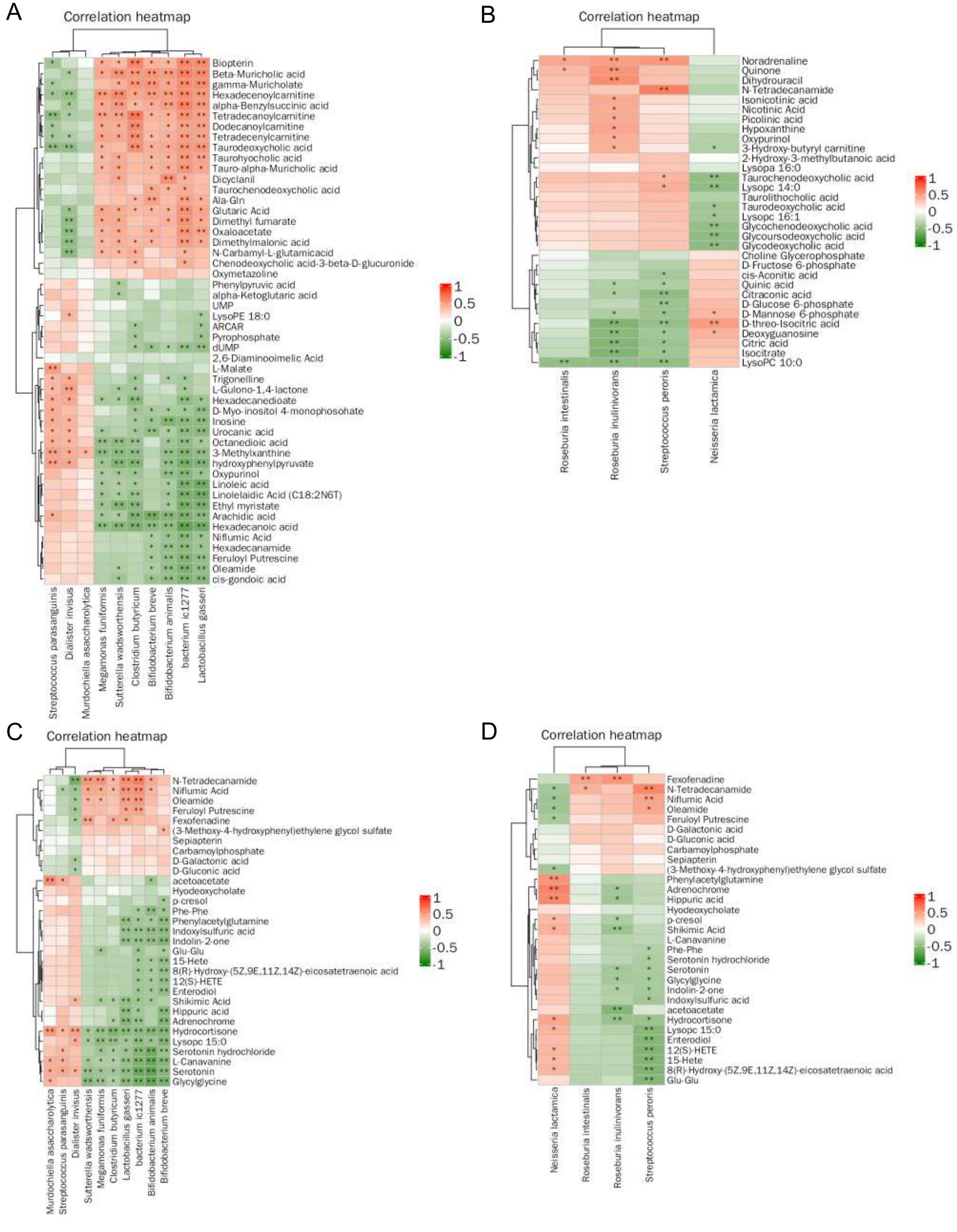
A
Correlation heatmap
Biopterin
Beta-Muricholic acid
gamma-Muricholate
Hexadecenoylcarnitine
alpha-Benzylsuccinic acid
Tetradecanoylcarnitine
Dodecanoylcarnitine
Tetradecenylcarnitine
Taurodeoxycholic acid
Taurohyocholic acid
Tauro-alpha-Muricholic acid
Dicyclanil
Taurochenodeoxycholic acid
Ala-Gln
Glutaric Acid
Dimethyl fumarate
Oxaloacetate
Dimethylmalonic acid
N-Carbamyl-L-glutamicacid
Chenodeoxycholic acid-3-beta-D-glucuronide
Oxymetazoline
Phenylpyruvic acid
alpha-Ketoglutaric acid
UMP
LysoPE 18:0
ARCAR
Pyrophosphate
dUMP
2,6-Diaminooimelic Acid
L-Malate
Trigonelline
L-Gulono-1,4-lactone
Hexadecanedioate
D-Myo-inositol 4-monophosohate
Inosine
Urocanic acid
Octanedioic acid
3-Methylxanthine
hydroxyphenylpyruvate
Oxypurinol
Linoleic acid
Linolelaidic Acid (C18:2N6T)
Ethyl myristate
Arachidic acid
Hexadecanoic acid
Niflumic Acid
Hexadecanamide
Feruloyl Putrescine
Oleamide
cis-gondoic acid
Streptococcus parasanguinis
Dialister invisus
Murdochiella asaccharolytica
Megamonas funiformis
Sutterella wadsworthensis
Clostridium butyricum
Bifidobacterium breve
Bifidobacterium animalis
bacterium ic1277
Lactobacillus gasseri
1
0.5
0
-0.5
-1
B
Correlation heatmap
Noradrenaline
Quinone
Dihydrouracil
N-Tetradecanamide
Isonicotinic acid
Nicotinic Acid
Picolinic acid
Hypoxanthine
Oxypurinol
3-Hydroxy-butyryl carnitine
2-Hydroxy-3-methylbutanoic acid
Lysopa 16:0
Taurochenodeoxycholic acid
Lysopc 14:0
Taurolithocholic acid
Taurodeoxycholic acid
Lysopc 16:1
Glycochenodeoxycholic acid
Glycoursodeoxycholic acid
Glycodeoxycholic acid
Choline Glycerophosphate
D-Fructose 6-phosphate
cis-Aconitic acid
Quinic acid
Citraconic acid
D-Glucose 6-phosphate
D-Mannose 6-phosphate
D-threo-Isocitric acid
Deoxyguanosine
Citric acid
Isocitrate
LysoPC 10:0
Roseburia intestinalis
Roseburia inulinivorans
Streptococcus peroris
Neisseria lactamica
1
0.5
0
-0.5
-1
C
Correlation heatmap
N-Tetradecanamide
Niflumic Acid
Oleamide
Feruloyl Putrescine
Fexofenadine
(3-Methoxy-4-hydroxyphenyl)ethylene glycol sulfate
Sepiapterin
Carbamoylphosphate
D-Galactonic acid
D-Gluconic acid
acetoacetate
Hyodeoxycholate
p-cresol
Phe-Phe
Phenylacetylglutamine
Indoxylsulfuric acid
Indolin-2-one
Glu-Glu
15-Hete
8(R)-Hydroxy-(5Z,9E,11Z,14Z)-eicosatetraenoic acid
12(S)-HETE
Enterodiol
Shikimic Acid
Hippuric acid
Adrenochrome
Hydrocortisone
Lysopc 15:0
Serotonin hydrochloride
L-Canavanine
Serotonin
Glycylglycine
Murdochiella asaccharolytica
Streptococcus parasanguinis
Dialister invisus
Sutterella wadsworthensis
Megamonas funiformis
Clostridium butyricum
Lactobacillus gasseri
bacterium ic1277
Bifidobacterium animalis
Bifidobacterium breve
1
0.5
0
-0.5
-1
D
Correlation heatmap
Fexofenadine
N-Tetradecanamide
Niflumic Acid
Oleamide
Feruloyl Putrescine
D-Galactonic acid
D-Gluconic acid
Carbamoylphosphate
Sepiapterin
(3-Methoxy-4-hydroxyphenyl)ethylene glycol sulfate
Phenylacetylglutamine
Adrenochrome
Hippuric acid
Hyodeoxycholate
p-cresol
Shikimic Acid
L-Canavanine
Phe-Phe
Serotonin hydrochloride
Serotonin
Glycylglycine
Indolin-2-one
Indoxylsulfuric acid
acetoacetate
Hydrocortisone
Lysopc 15:0
Enterodiol
12(S)-HETE
15-Hete
8(R)-Hydroxy-(5Z,9E,11Z,14Z)-eicosatetraenoic acid
Glu-Glu
Neisseria lactamica
Roseburia intestinalis
Roseburia inulinivorans
Streptococcus peroris
1
0.5
0
-0.5
-1

Figure S5 Correlations between species-level microbiota and metabolites in PAH-CHD children. **A**, Correlation between gut microbiota and metabolites. **B**, Correlation between pulmonary microbiota and metabolites. **C**, Correlation between gut microbiota and blood metabolites. **D**, Correlation between pulmonary microbiota and blood metabolites. Spearman correlation analysis was used to determine correlations. Blue indicates positive correlation, orange indicates negative correlation. Wilcoxon rank-sum test was used, with multiple testing correction by Benjamini-Hochberg false discovery rate (FDR; $P<0.05$). *$P<0.05$, **$P<0.01$, and ***$P<0.001$.

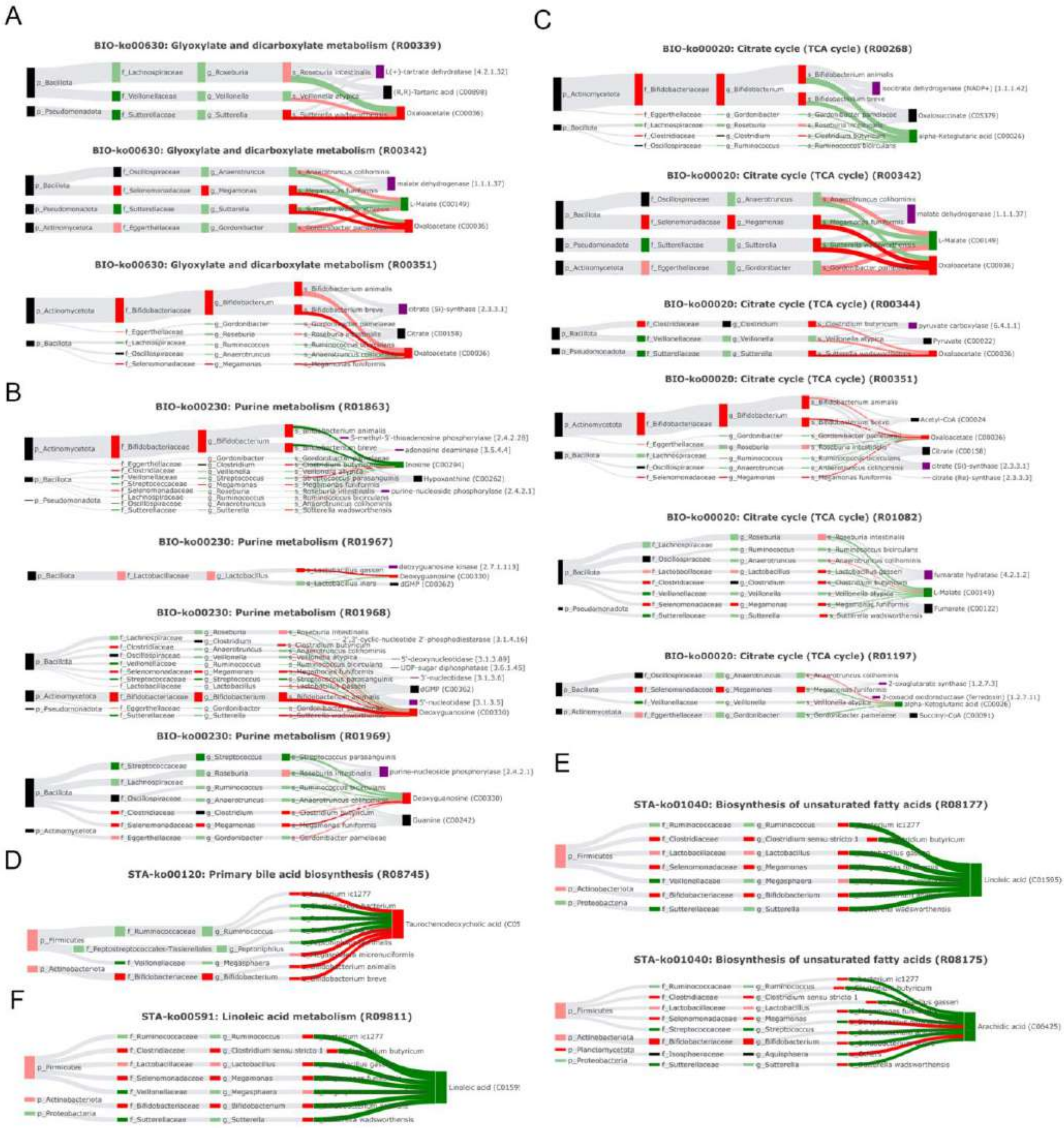

Figure S6 Characteristic associations between gut microbiota and metabolites. **A**, BIO-ko00630: Glyoxylate and dicarboxylate metabolism.**B**, BIO-ko00230: Purine metabolism. **C**, BIO-ko00020: Citrate cycle (TCA cycle). **D**, STA-ko00120: Primary

bile acid biosynthesis. **F**, STA-ko00591: Linoleic acid metabolism. **E**, STA-ko01040: Biosynthesis of unsaturated fatty acids. H, Network summary analysis diagram of gut microbiota, metabolites, and metabolic pathways. In the BIO-Sankey and STA-Sankey network diagrams, dark red (or green) bars indicate significantly higher (or lower) levels of microorganisms or metabolites in high-altitude populations ($|FC| > 1$, $P < 0.05$); light red (or green) bars indicate microorganisms or metabolites that are higher (or lower) in high-altitude populations ($|FC| > 1$, $P \geq 0.05$); black bars represent microorganisms or metabolites from reference databases; purple bars indicate metabolic enzymes; dark red (or green) strips represent significantly positive (or negative) correlations (Spearman correlation test, $|R| > 0$, $P < 0.05$); light red (or green) strips indicate non-statistically significant positive (or negative) correlations (Spearman correlation test, $|R| > 0$, $P \geq 0.05$); gray strips represent reference relationships retrieved from databases. FC refers to fold change, which is the ratio of the average value of all biological replicates of each metabolite in the control group; P-values are calculated using a Student's t-test and represent the level of significant differences. The related analyses were performed based on the MetOrigin platform.

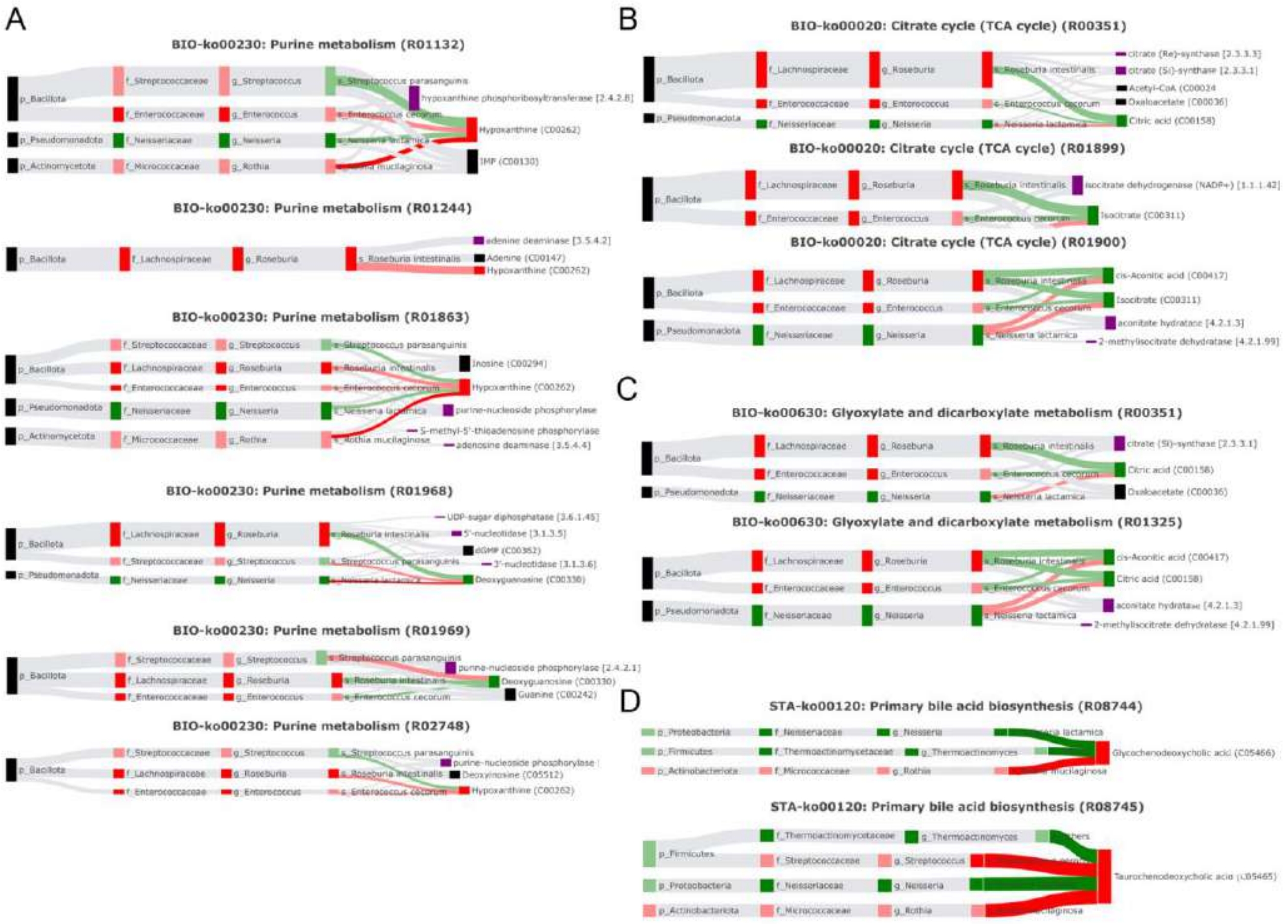

Figure S7 Characteristic associations between lung microbiota and metabolites. **A**, BIO-ko00230: Purine metabolism .**B**, BIO-ko00020: Citrate cycle. **C**, BIO-ko00630: Glyoxylate and dicarboxylate metabolism. **D**, STA-ko00120: Primary bile acid biosynthesis. In the BIO-Sankey and STA-Sankey network diagrams, dark red (or green) bars indicate significantly higher (or lower) levels of microorganisms or metabolites in high-altitude populations (|FC| > 1, P < 0.05); light red (or green) bars indicate microorganisms or metabolites that are higher (or lower) in high-altitude populations (|FC| > 1, P ≥ 0.05); black bars represent microorganisms or metabolites

from reference databases; purple bars indicate metabolic enzymes; dark red (or green) strips represent significantly positive (or negative) correlations (Spearman correlation test, $|R| > 0$, $P < 0.05$); light red (or green) strips indicate non-statistically significant positive (or negative) correlations (Spearman correlation test, $|R| > 0$, $P \geq 0.05$); gray strips represent reference relationships retrieved from databases. FC refers to fold change, which is the ratio of the average value of all biological replicates of each metabolite in the control group; P-values are calculated using a Student's t-test and represent the level of significant differences. The related analyses were performed based on the MetOrigin platform.

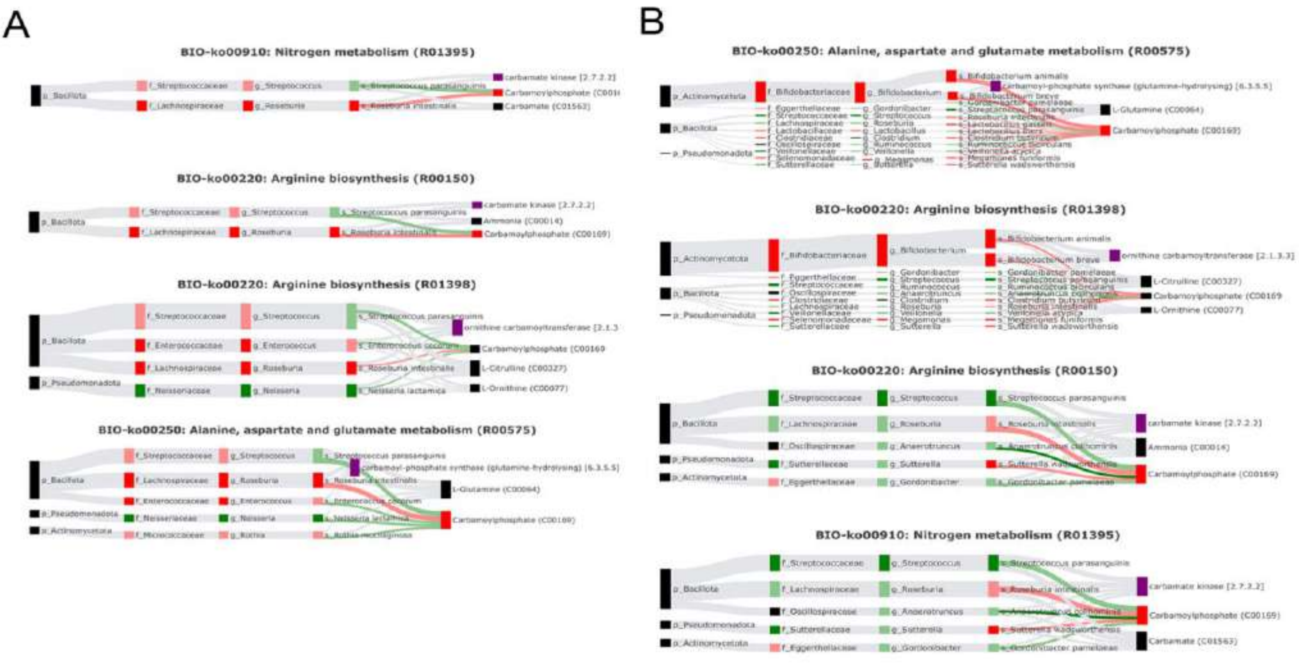

Figure S8 Characteristic associations between gut and lung microbiota and blood metabolites. **A**, BIO-Sankey network diagram of lung microbiota and blood metabolites. **B**, BIO-Sankey network diagram of gut microbiota and blood metabolites. In the BIO-Sankey network diagrams, dark red (or green) bars indicate significantly higher (or lower) levels of microorganisms or metabolites in high-altitude populations ($|FC| > 1$, $P < 0.05$); light red (or green) bars indicate microorganisms or metabolites that are higher (or lower) in high-altitude populations ($|FC| > 1$, $P \geq 0.05$); black bars represent microorganisms or metabolites from reference databases; purple bars indicate metabolic enzymes; dark red (or green) strips represent significantly positive (or negative) correlations (Spearman correlation test, $|R| > 0$, $P < 0.05$); light red (or green) strips indicate non-statistically

significant positive (or negative) correlations (Spearman correlation test, $|R| > 0$, $P \geq 0.05$); gray strips represent reference relationships retrieved from databases. FC refers to fold change, which is the ratio of the average value of all biological replicates of each metabolite in the control group; P-values are calculated using a Student's t-test and represent the level of significant differences. The related analyses were performed based on the MetOrigin platform.
